# Effectiveness and Safety of Traditional Chinese Medicine in Treating COVID-19: Clinical Evidence from China

**DOI:** 10.14336/AD.2021.0906

**Published:** 2021-12-01

**Authors:** Dongmei Xing, Zhibin Liu

**Affiliations:** ^1^Department of Cardiovascular Disease Diagnosis and Treatment Center, the First Affiliated Hospital of Henan University of Chinese Medicine, Zhengzhou, China.; ^2^Department of AIDS Treatment and Research Center, the First Affiliated Hospital of Henan University of Chinese Medicine, Zhengzhou, China.; ^3^Henan Key Laboratory of Viral Diseases Prevention and Treatment of Traditional Chinese Medicine, the First Affiliated Hospital of Henan University of Chinese Medicine, Zhengzhou, China

**Keywords:** COVID-19, Western medicine (WM), Traditional Chinese Medicine (TCM), signs and symptoms, overall mortality

## Abstract

When the outbreak of COVID-19 occurred in 2020, the Chinese government promptly undertook a series of preventive control and medical treatment measures that have effectively reduced the infection and mortality rates of COVID-19. In the process of preventing COVID-19 transmission and treating COVID-19, the Chinese government actively promotes the use of traditional Chinese medicine (TCM), and a series of studies have been carried out to determine the efficacy of TCM for COVID-19. Chinese physicians have accumulated rich experiences and created three Chinese patent medicines (i.e., *Lianhua Qingwen* Capsule, *Jinhua Qinggan* Granules, *and Xuebijing* Injection) and three herbal prescriptions (i.e., *Xuanfeibaidu Recipe, Huashi Paidu Recipe, and Qingfei Paidu* Decoction), as well as other strategies based on TCM theory to effectively treat COVID-19. Studies have reported that TCM treatment plays a significant role in improving the clinical symptoms, shortening the duration of hospitalization, reducing the overall mortality rate, obtaining favorable treatment outcomes in patients with severe COVID-19, preventing disease progression, improving quality of life and immunity, and reducing the positive rate of viral nucleic acid testing. TCM treatment has a fairly high degree of safety, but the level of evidence needs to be further improved.

Severe acute respiratory syndrome coronavirus 2 (SARS-CoV-2) is caused by the new coronavirus that was first recognized by Chinese scientists in January 2020 [1]. SARS-Co-V-2 causes coronavirus disease (COVID-19), which has a potent transmission, fast infection rate, diverse clinical manifestations, and a relatively high mortality rate. On January 30, 2020, the Director-General of the WHO declared the novel coronavirus outbreak a public health emergency of international concern, which is the highest level of alarm of the WHO. By August 31, 2021, the COVID-19 pandemic caused by SARS-CoV-2 had led to nearly 216 million cases and just under 4.5 million deaths worldwide (www.who.int/publications/m/item/weekly-epidemiological-update-on-covid-19---31-august-2021).

Since January 2020, the Chinese government has promptly implemented a series of preventive control and medical treatment measures, including a cooperative treatment strategy comprising Western medicine (WM) (i.e., conventional medicine) and traditional Chinese medicine (TCM) that has effectively reduced the infection and mortality rates of COVID-19. In the process of preventing the transmission of SARS-CoV-2 and treating COVID-19, Chinese physicians have actively conducted studies that have resulted in the identification of three Chinese patent medicines (i.e., *Lianhua Qingwen* Capsule, *Jinhua Qinggan* Granules, and *Xuebijing* (XBJ) Injection) and three herbal prescriptions (i.e., *Xuanfeibaidu Recipe, Huashi Paidu Recipe,* and *Qingfei Paidu* Decoction (QFPDD)) based on TCM theory to effectively control the COVID-19 outbreak (www.nhc.gov.cn/xcs/zhengcwj/202003/46c9294a7dfe4cef80dc7f5912eb1989.shtml). On March 2, 2021, the State Medical Products Administration of China approved the listing of *Qingfei Baidu* Granules produced by the Institute of Clinical Basic Medicine of China Academy of Chinese Medical Sciences, *Hua-Shi-Baidu* Granules produced by Guangdong Yifang Pharmaceutical Co., Ltd., and *Xuanfei Baidu* Granules produced by Shandong Buchang Pharmaceutical Co., Ltd.

In China, both WM and TCM are considered mainstream medicines. As COVID-19 is an emerging infectious disease, there are some differences between TCM and WM regarding COVID-19 treatment and evaluation strategies. However, recent research has suggested that TCM and WM share similar philosophical approaches to fighting this disease [2]. The present article summarizes the progress of TCM treatment of COVID-19 based on clinical studies in China.

## Alleviation of COVID-19-related signs and symptoms

The signs and symptoms of COVID-19 result from conditions that do not normally develop in individuals with healthy immune systems. Fever, fatigue, and cough are the most common symptoms experienced by patients with COVID-19. A meta-analysis showed that 88.7% of more than 660 patients with confirmed COVID-19 present with fever [3], and a WHO review found that 87.9% of more than 55,000 Chinese patients with COVID-19 had fever (www.nhc.gov.cn/jkj/s3578/202002/87fd92510d094e4b9bad597608f5cc2c.shtml). The reported prevalence of cough is 81% in 62 patients with COVID-19 in Zhejiang Province [4], while the reported prevalence of fatigue is 75% [5]. The persistence of these COVID-19 symptoms leads to increased incidences of anxiety and depression, decreased treatment compliance, reduced quality of life (QOL), and worsening of the disease. Chinese medical professionals often use TCM syndrome differentiation through the combination of four diagnostic methods. TCM has been proved to improve the clinical symptoms of many diseases. Clinical observation of patients with novel coronavirus pneumonia shows that TCM rapidly relieves the symptoms of COVID-19.

Ba et al. [6] reported the effectiveness of the administration of 200 ml of *pneumonia no. 1 formula* (comprising *Bupleurum chinense, Scutellaria baicalensis Georgi, Pinellia ternata, Codonopsis pilosula, Trichosanthes kirilowii Maxim, Areca catechu, Amomum tsaoko Crevost et Lemarie, Magnolia officinalis Rehd, Anemarrhena asphodeloides, Paeonia lactiflora, Glycyrrhizae, tangerine*, and *Polygonum cuspidatum*) twice daily to 451 patients with confirmed COVID-19. This treatment almost completely resolved the symptoms of fever, cough, and fatigue, and improved other symptoms, such as nausea. Furthermore, the lung lesions of 415 patients (92.02%) were reduced compared with the first lung CT scan.

Duan et al. [7] randomized 123 patients with confirmed COVID-19 into a treatment group and a control group. The control group were treated with WM, while the treatment group were treated with WM plus *jinhua qinggan granules* (comprising *Lonicera japonica, Gypsum fibrosum, Ephedra sinica Stapf, Semen Armeniacae amarum, Scutellaria baicalensis Georgi, Forsythia suspensa, Fritillaria, Anemarrhena asphodeloides, Fructus arctii, Artemisia carvifolia, Mentha haplocalyx,* and *Glycyrrhiza uralensis Fisch)*. After 5 days of treatment, the fever, cough, and fatigue had disappeared in 80.3%, 66.1%, and 77.6% of patients, respectively, in the treatment group; and 53.1%, 42.9%, and 53.8%, respectively, in the control group. The results demonstrate that *jinhua qinggan granules* effectively improve the clinical symptoms of COVID-19.

Wang et al. [8] treated 55 patients according to their wishes at the beginning of hospitalization for COVID-19. The 23 patients in the TCM group were treated with *Huashi Baidu* Granules combined with intravenous injections of *Xiyanping* (100 mg twice daily), *XBJ* (100 ml twice daily), and Shenmai (60 ml four times daily) to treat the TCM syndrome of an epidemic toxin blocking the lung. The 32 patients in the WM group received antiviral therapy, antibiotics, and corticosteroid therapy. There was no pre-treatment difference between the two groups in the imaging features, except for the distribution areas. After treatment, the imaging scores for total distribution and for the left upper lung were significantly lower in the TCM group than the WM group, which indicates that TCM effectively promotes the resolution of inflammation.

## Shortening of the duration of hospitalization

The duration of hospitalization is related to many factors, including the severity of COVID-19, morbidity, age, time delay after admission before receiving TCM treatment, and TCM treatment duration [9]. The reported duration of hospitalization varies between different time periods and studies. In February 2020, it was reported that the average hospital stay was approximately 9 days for 632 patients in China, 20 days for 396 patients in Hubei Province, 12.75 days for 20 patients in Guangdong Province, and 5 days for four patients in Hainan Province (www.nhc.gov.cn/xcs/fkdt/202002/35990d56cfcb43f4a70d7f9703b113c0.shtml). In May 2020, the average duration of hospitalization of 577 discharged patients with confirmed COVID-19 in Beijing was 25 days, which is longer than that reported during the initial months of the COVID-19 outbreak (www.nhc.gov.cn/xcs/fkdt/202005/9e0a7a85ee494b8d9f26be2645c1f12c.shtml). Various studies have revealed that the average duration of hospitalization for COVID-19 was 10 days for 138 patients in Hubei Province [10], 13 days for 77 patients in Beijing [11], 14 days for 91 patients in Hainan [12], and 12.8 days for 1,099 patients in China [13]. Patients with COVID-19 with a longer duration of hospitalization use more medical resources, resulting in higher medical costs. Therefore, shortening the hospital stay will significantly reduce the costs of this serious disease [14].

Studies have found that TCM effectively shortens the duration of hospitalization of patients with COVID, irrespective of the severity of disease. Shi et al. [15] observed 782 patients with COVID-19 receiving QFPDD in 54 hospitals in nine provinces of China and found that patients who received early treatment with QFPDD within 1 week, 1-2 weeks, or 2-3 weeks had a higher likelihood of recovery. The median course of the disease decreased from 34 days to 24 days, 21 days, and 18 days in accordance with the administration of QFPDD treatment at 2-3 weeks after admission, 1-2 weeks after admission, and within 1 week of admission, respectively. QFPDD treatment within 1 week was related to a decrease of 1-4 days in the median duration of hospital stay compared with later QFPDD treatment. These findings suggest that early treatment with QFPDD is associated with a shorter hospital stay.

## Reduction of overall mortality

The China-WHO joint investigation into coronavirus pneumonia (COVID-19) indicated that the mortality rate in China was 3.8% and increased with age, with the highest mortality rate being 21.9% for patients older than 80 years (www.nhc.gov.cn/jkj/s3578/202002/87fd92510d094e4b9bad597608f5cc2c.shtml). The overall mortality rate of COVID-19 is fairly high because COVID-19 has potentially fatal complications, including acute respiratory distress syndrome (ARDS), acute renal injury, acute cardiac injury, acute neurologic disease, thrombosis, infections, and shock. Mortality is an important index to measure the impact of an epidemic. The mortality is directly related to the severity of the disease and is reduced by early intervention and effective treatment.

A retrospective cohort study evaluated 282 severely and critically ill patients with COVID-19 who were diagnosed, typed, and basically treated in accordance with *the 7th edition of the Diagnosis and Treatment Guidelines for COVID-19 (Trial Version)* [16]. TCM treatment was administered to 186 patients (66.0%) (i.e., one to five of the drugs that are contained in *Maxingshigan Decoction*), but not to the other 96 patients (34.0%) (non-TCM treatment group). A propensity score-matched analysis found that the overall mortality of patients with severe COVID-19 was significantly lower in the TCM treatment group than the non-TCM treatment group (21.3% [20/94] vs. 39.4% [37/94]). Multivariable logistic regression showed that the severity and treatment duration of TCM were associated with the all-cause mortality of COVID-19. This study suggests that TCM treatment may reduce the mortality rate of patients with severe/critical COVID-19.

Chen et al. [17] collected data from 662 in-patients with COVID-19 in Wuhan before March 20, 2020, including 562 (84.9%) with severe disease and 100 (15.1%) with critical disease. All patients were diagnosed, typed, and basically treated in accordance with *the 5th edition of the Diagnosis and Treatment Guidelines for COVID-19 (Trial Version)*. Patients were divided into a TCM-WM group who received *Mahuang LiuJun Tang* plus WM, and a WM group who received only WM. The respective number (prevalence) of severe and critical patients was 444 (91.7%) and 40 (8.3%) in the TCM-WM group, and 118 (66.3%) and 60 (33.7%) in the WM group. Fifteen patients (3.1%) in the TCM-WM group and 56 patients (31.5%) in the WM group died. After propensity score matching in a 1:1 ratio, the all-cause mortality was lower in the TCM-WM group (8.33%, 13/156) than the WM group (23.08%, 36/156). After multivariate adjustment, the mortality risk of the TCM group was reduced by 82.2% compared with the WM group, which suggests that TCM significantly reduces the mortality of COVID-19.

Another study of 8,939 patients from 15 hospitals in China reported an overall in-hospital COVID-19-related mortality of 3.7% [18]. The COVID-19-related mortality was 1.2% among 2,568 patients receiving qingfei paidu tang (QPT) and 4.8% among 6,371 patients not receiving QPT. After adjustment for patient characteristics and concomitant treatments, QPT use was associated with a 50% reduction of in-hospital COVID-19-related mortality.

## Favorable treatment outcomes in patients with severe COVID-19

Patients with severe COVID-19 are more likely to develop septic shock, multiple organ dysfunction syndrome, irreversible coagulation disorders, uncorrectable metabolic acidosis, and ARDS than those with mild COVID-19. The mortality rate and medical cost of severe and critically ill patients with COVID-19 are higher than those of patients with mild and moderate COVID-19. Therefore, a favorable treatment efficacy for severe and critically ill patients with COVID-19 helps to reduce the mortality rate and saves medical resources.

Luo et al. [19] equally randomized 60 patients with severe COVID-19 into the XBJ group and the control group; after three patients dropped out, the final cohort comprised 29 patients in the XBJ group and 28 in the control group. After 2 weeks of treatment, the respective rates of mechanical ventilation in the XBJ group and the control group were 10.34% and 46.42%. Compared with the control group, the XBJ group had a lower incidence of septic shock (6.9% vs. 28.57%) and shorter length of ICU stay (8.42 days vs. 10.72 days). Furthermore, the number (incidence) of patients with severe COVID-19 who were downgraded to moderate cases was 25 (86.2%) in the XBJ group and 18 (64.3%) in the control group, and the proportion of patients with severe COVID-19 who became critically ill was lower in the XBJ group (10.3%) than the control group (35.7%). These findings suggest that XBJ significantly promotes the conversion of severe COVID-19 to moderate and reduces the rate of conversion from severe to critical disease.

Another study randomly assigned 111 patients with severe and critical COVID-19 to the *Shenhuang* Granule (SHG) group and the control group [20]. After 2 weeks of treatment, the mortality rate was lower in the SHG group (38.6%, 22/57) than the control group (75.9%, 41/54). A post hoc analysis of the patients with severe COVID-19 found that the mortality rate was lower in the SHG group (58.8%, 10/17) than the control group (5.3%,1/19). A similar trend was seen in the cohort of patients with critical disease, with a mortality rate of 55.3% (21/38) in the SHG group compared with 83.8% (31/37) in the control group. These results suggest that SHG is a promising therapy that significantly reduces the mortality rate of patients with severe and critical COVID-19.

## Prevention of COVID-19 progression

The China-WHO joint investigation into COVID-19 reported that 13.8% of patients had severe illness (www.nhc.gov.cn/jkj/s3578/202002/87fd92510d094e4b9bad597608f5cc2c.shtml), and the mortality rate of patients with severe COVID-19 is as high as 50%. Another study showed that about 20% of patients with COVID-19 may develop severe disease with a high risk of mortality [21]. Therefore, it is important to prevent disease progression to reduce mortality.

A study of patients with COVID-19 treated with TCM in Jiangxia Cabin Hospital compared with those treated with WM in Qiaokou Cabin Hospital found that no patients got worse in Jiangxia Cabin hospital, while 10% of patients in Qiaokou Cabin Hospital had their condition aggravated [22]. This suggests that TCM effectively prevents COVID-19 progression.

Tian et al. [23] observed 721 patients with mild and moderate COVID-19 (including 430 patients in the *Hanshiyifang* (HSYF) group and 291 in the control group) and found that no patients in the HSYF group progressed to severe disease, while the condition was aggravated in 19 patients (6.55%) in the control group; this suggests that HSYF effectively reduces the rate of conversion to severe disease in patients with mild and moderate COVID-19. Hu et al. [24] randomized 300 patients with confirmed mild to moderate COVID-19 into the *Jinyinhua* oral liquid group and the WM group and found that the rate of conversion to severe disease was 0% in the *jinyinhua* oral liquid group and 4.22% in the WM group; this suggests that TCM prevents aggravation of COVID-19 and relatively reduced the incidence of severe COVID-19 by 100%. A meta-analysis also obtained similar findings [25].

## Improved quality of life

An increasing number of patients with confirmed COVID-19 are entering the recovery stage as the outbreak becomes more controlled. Timely and effective treatment reduces the incidence of complications, which coincides with the TCM concepts of ‘reducing the incidence of disease’ and ‘preventing disease progression’. Pulmonary fibrosis is a common complication of COVID-19 due to poor absorption of pulmonary inflammation, causing a pulmonary function decline of 20%-30% and dyspnea, which decreases the QOL. The TCM syndromes Qi deficiency, Yin deficiency, and Yang deficiency are common in the recovery stage of COVID-19. The potential TCM treatments for these conditions are *Shengmai* powder, *Qingzao jiufei* decoction, and *Liujunzi* decoction.

Attention must also be paid to the psychological impact of COVID-19. Treatments such as TCM, *Baduanjin*, *Taijiquan*, ear acupressure, acupuncture, and moxibustion should be applied to improve physical and psychological health and QOL [26].

## Improved immunity

Immunity is necessary to eliminate pathogenic bacteria, resist external infection, and maintain internal homeostasis. Immunity indicators directly reflect the patient's body state. TCM improves the immunity, prevents the progress of disease, and leads to recovery.

Fu et al. [27] performed a randomized controlled trial of patients with COVID-19 treated with integrated TCM (*Toujie Quwen* Granules) or WM (Abidole and ambroxol hydrochloride). After treatment, the TCM group had a greater increase in the lymphocyte count and greater decrease in the C-reactive protein level than the WM group. In the early stage, the CD4+ T cell counts in the WM group decreased after an initial upregulation, while the expressions of CD8+ and CD45+ in the TCM group remained stable after the initial upregulation. Overall, the expressions of CD4+ and CD8+ increased in the TCM group and decreased in the WM group. These findings suggest that TCM controls the expressions of T cells and restores the immune function.

A retrospective study analyzed the efficacy of QFPDD in reducing the levels of inflammatory cytokines in patients with COVID-19 [28]. Twenty-four critically ill patients were treated with conventional WM alone, while 12 were treated with QFPDD plus WM. The inflammatory indicators were improved in both groups, and the levels of inflammatory cytokines were effectively reduced. Compared with WM alone, the administration of WM plus QFPDD significantly reduced the levels of C-reactive protein, interleukin (IL)-2R, IL-6, IL-10, tumor necrosis factor-α, and other inflammatory factors, improved the rate of body temperature recovery, improved the CT findings, increased the conversion from critical to normal and mild disease, and reduced the mortality rate and incidences of other clinical conditions.

Chen et al. [29] evaluated the effectiveness of XBJ in the treatment of severe and critical COVID-19. The XBJ group had improved respiratory function and immunity after treatment and had better improvement of the ratio of CD4+ T cells than the conventional treatment group. This suggests that XBJ improves lung function and immunity.

## Reduction of the positive rate of viral nucleic acid testing

The positive rate of RT-PCR detection of SARS-CoV-2 in patients discharged from hospital after COVID-19 recovery is concerning, with a reported range of 5% to 15% (www.nhc.gov.cn/xcs/fkdt/202005/dd9e76dabf154c1cafc8dba40c57eac9.shtml). If this positive rate continues to grow, it may lead to a repeat outbreak of COVID-19.

He et al. [30] observed 420 patients with COVID-19 and found that symptoms such as fatigue, cough, and insomnia were improved in the TCM-WM group. Furthermore, the positive rate of SARS-COV-2 nucleic acid testing after meeting the discharge criteria was 2.8% (9/325) in the TCM-WM group and 15.8% (15/95) in the WM group. This suggests that TCM helps to reduce the positive rate of RT-PCR detection of SARS-CoV-2 in discharged patients with confirmed COVID-19. TCM was considered an independent factor that reduced the positive rate of SARS-CoV-2 nucleic acid testing.

## Treatment safety

Certain popular TCM herbs have adverse or toxic effects, including *Ephedra sinica (ma huang)* and *Asarum (xi xin). E. sinica* reportedly causes hepatic, renal, and cardiologic toxicity due to its high content of ephedrine, while *Asarum* may cause renal and carcinogenic toxicity due to its high content of aristolochic acid [31]. In clinical practice, TCM specialists believe that the incidences of these adverse reactions can be reduced or avoided with reasonable compatibility, correct processing, and appropriate dosage in accordance with the TCM method and their clinical experiences. During the COVID-19 pandemic, TCM practitioners have made detailed records of the possible adverse effects of TCM herbs. The common adverse reactions are nausea and vomiting, diarrhea, liver damage, and a reduced white blood cell count. Studies have showed that Chinese patent medicines (such as *XBJ* injection, *lianhuaqingwen* capsules, and *Shufeng Jiedu* Capsules) and TCM prescriptions (such as Q-14 (*qingwen baidu Recipe*), *pneumonia no. 1 formula*, and *QingFei TouXie FuZheng Fang*) do not cause adverse reactions; these findings confirm the safety of TCM in the treatment of COVID-19 (www.gov.cn/xinwen/2020-03/23/content_5494694.htm). A meta-analysis obtained similar conclusions [32].

Zhou et al. [20] evaluated patients with severe and critical COVID-19 and found that the administration of SHG significantly reduced the incidence of adverse events, such as an increased blood sugar level, increased blood total bilirubin level, thrombocytopenia, respiratory failure or ARDS, cardiopulmonary failure, and multiple organ dysfunction syndrome. This study suggests that treatment with SHG is associated with an increased lymphocyte count and decreased incidence of adverse events.

Adverse reactions to jinhua qinggan granules have been reported. Duan et al. [7] reported that 27 patients (32.93%) with mild or moderate COVID-19 had diarrhea after taking jinhua qinggan granules; eight of these patients got better after stopping the treatment, while the other 19 patients got better without the need for any antidiarrheal drugs. The safety of TCM in the treatment of COVID-19 needs further study.

## Conclusions

Based on TCM theory and clinical syndrome differentiation to formulate a treatment plan, timely TCM treatment strives to effectively control COVID-19. The National Health Commission of the People’s Republic of China and the National Administration of TCM continually update the diagnosis and treatment of COVID-19 pneumonia and have established a TCM treatment schedule and joint TCM and WM strategy to improve the treatment effectiveness. The Ministry of Science and Technology of the People’s Republic of China project urgently assigns studies to investigate the prevention and treatment of COVID-19. Relevant departments of provinces and cites also strongly support the implementation of studies related to COVID-19 to explore the characteristics, clinical manifestations, prognosis, and treatment of the disease, screen potential drugs, and develop vaccines.

Chinese physicians have actively explored clinical treatment plans and carried out clinical studies on COVID-19 that have proved the effectiveness and safety of TCM in treating COVID-19 from different perspectives. Although there are some problems such as ambiguity and clinical design defects, we believe that additional research will reveal the importance of TCM in the treatment of COVID-19.
